# Arterial Suture

**Published:** 1890-07-12

**Authors:** 


### SURGERY.

### ARTERIAL SUTURE.

"Arterial Suture, an Experimental Study," is the title of the inaugural or graduation thesis presented by Dr. Jassinowski before the University of Dorpat. It does not appear to have, as yet, attracted the notice of the medical press in this country, but is certainly one of the most remarkable contributions to modern surgery. From the time of Ambrose Paré to the present day the idea of repairing a wound in an artery has been deemed Utopian, but Dr. Jassinowski has shown by a long series of experiments on animals that within certain limits the operation is perfectly feasible. True, in by far the larger proportion of cases the old practice of completely severing the vessel and ligaturing it above and below, trusting to the establishment of a collateral circulation, will continue to be followed, but there are some arteries, as the iliac and subclavian, the carotids, and others, where it would be highly desirable to maintain the normal course of the circulation, and, above all, the popliteal, injury of which at present necessarily involves the loss of the leg, collateral circulation being impossible between the parts of the limb above and below the knee. Dr. Jassinowski has shown (1) that by his procedure union may be obtained by first intention; (2) that hæmorrhage may be entirely obviated during the operation; (3) that secondary hæmorrhage need not be feared; (4) that it is indicated in all cases in which the wound is fresh and clean, longitudinal, oblique, or in the form of a flap; (5) and in which not more than half the circumference of the vessel is involved; (6) that the strictest antiseptic precautions are essential; and (7) that under these conditions the operation is by no means difficult. His procedure is (1) to grasp the vessel above the wound with a lever clamp or forceps; (2) to expose the artery at the seat of injury, the sheath being at the same time pushed aside; (3) to pass the sutures continuously and in a horizontal direction through the outer and middle coats only of the artery; (4) to secure the thread merely by a double knot at each end, cutting it off short at each; (5) to remove the clamp, exerting gentle pressure on the artery during the restoration of the circulation through it; (6) to unite the margins of the sheath by a similar suture; (7) to unite by suture the fascia and the skin; and (8) lastly to seal the outer wound with collodion. The sutures for the vessel, sheath, and fascia should of course be of sterilised catgut, those used for closing the cutaneous wound may be of silver.

